# Polynucleotide injection treatment for iatrogenic fat atrophy in two patients: Potential for safe volumization in aesthetic medicine

**DOI:** 10.1111/srt.13439

**Published:** 2023-08-13

**Authors:** Michael James Kim, Hyun‐Jun Park, Seung Min Oh, Kyu‐Ho Yi

**Affiliations:** ^1^ Aeon Medical and Aesthetic Centre Singapore Singapore; ^2^ Maylin Clinic (Chungdam) Seoul Republic of Korea; ^3^ Maylin Clinic (Apgujeong) Seoul Republic of Korea; ^4^ Division in Anatomy and Developmental Biology, Department of Oral Biology, Human Identification Research Institute, BK21 FOUR Project Yonsei University College of Dentistry Seoul Republic of Korea

Dear Editor,

1

The current trend in rejuvenation techniques has shifted from direct application of synthetic substances—such as collagen, hyaluronic acid, and glycoproteins—to the dermal layer, to a more cell‐centric approach that stimulates the skin's cellular components, particularly fibroblasts.

Using purified polynucleotides (PN) from fish germ cells, recent advancements in autologous substances stimulate skin cell replication and metabolic activity, promoting tissue regeneration and a more natural renewal process than traditional fillers.

PN injections have found widespread application in various clinical scenarios, including improving skin elasticity, thickness, wrinkles, hydration, pore size, and pigmentation. While most clinical outcomes have focused on dermal rejuvenation, polydeoxyribonucleotides (PDRNs) have demonstrated the ability to stimulate pre‐adipocyte proliferation in vitro.^1^ Furthermore, high‐viscosity PN injections have been utilized for the treatment of hypertrophic, atrophic, surgical, and different types of acne scars.^2^, ^3^ PDRNs, derived from the identical species as PN, have shown effectiveness in treating wounds and ulcers.^4^ As an adenosine A_2A_ receptor agonist and because of their role in the salvage pathway for damaged DNA, they have gained recognition for its rejuvenational and biochemical properties in aesthetic medicine.^5^, ^6^ However, there are much less studies on PNs and their role in tissue rejuvenation.

The technology used to produce PNs differs just as the cross‐linking technology determines stark differences in dermal hyaluronic fillers. Studies on PN have been published previously in the treatment of periorbital rhytides^7^ and post‐surgery scars^2^; however, this is the first study using PN as a volumizing treatment for fat atrophy in vivo. Although various dermal fillers, especially hyaluronic acid fillers, are commonly used for volume replacement in the face, serious and non‐reversible complications—such as skin necrosis and retinal artery occlusion—harken the need for a safer yet effective alternative.^8^ This study delves into the impact of administering PN injections into the subcutaneous layer to restore iatrogenic volume loss from lipolysis injections.

Under this evidence, PN was used to treat two patients with areas of volume loss on their temple and cheek, respectively.

## 2 CASE 1

**FIGURE 1:**
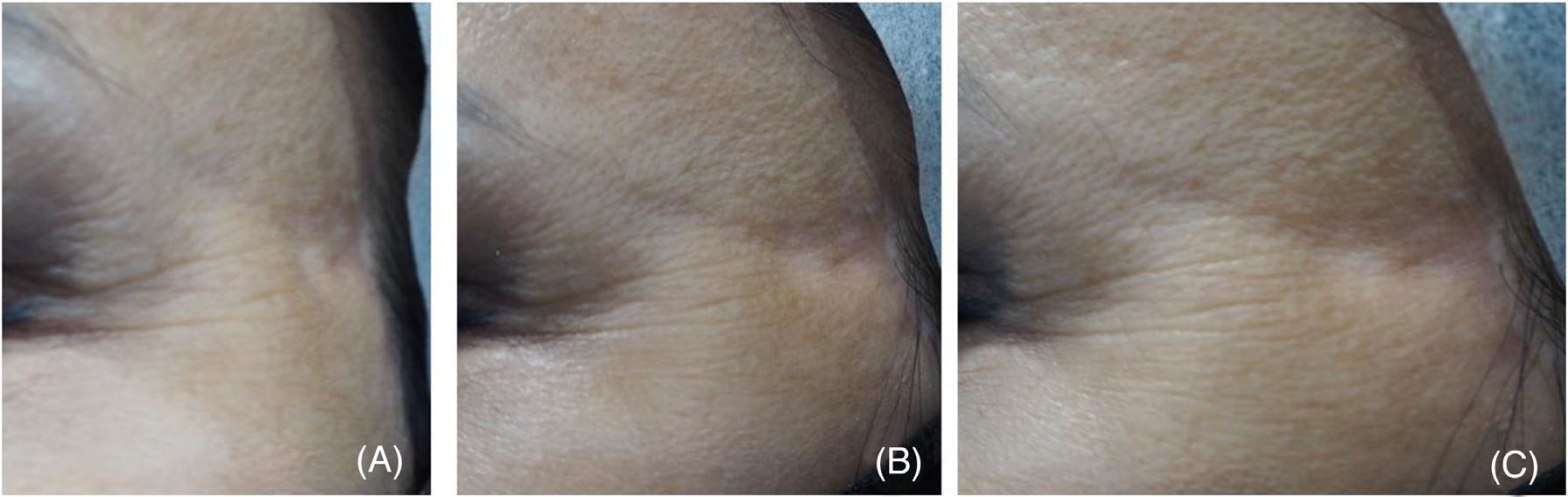
A 53‐year‐old woman underwent lipolysis treatment and developed an undesired localized depression in the left temple region following initial treatment. Patient images are shown from frontal (A), 45° (B), and lateral views (C).

A 53‐year‐old East Asian female received lipolysis injection treatment in South Korea 14 months ago to reduce facial fat, mainly in the jowls and temples. However, she developed an undesired depression in her left temple area in a triangular shape, extending to the lateral end of her left eyebrow (Figure 1), with no skin changes like thinning, erythema, or texture difference compared to surrounding skin.

**FIGURE 2:**
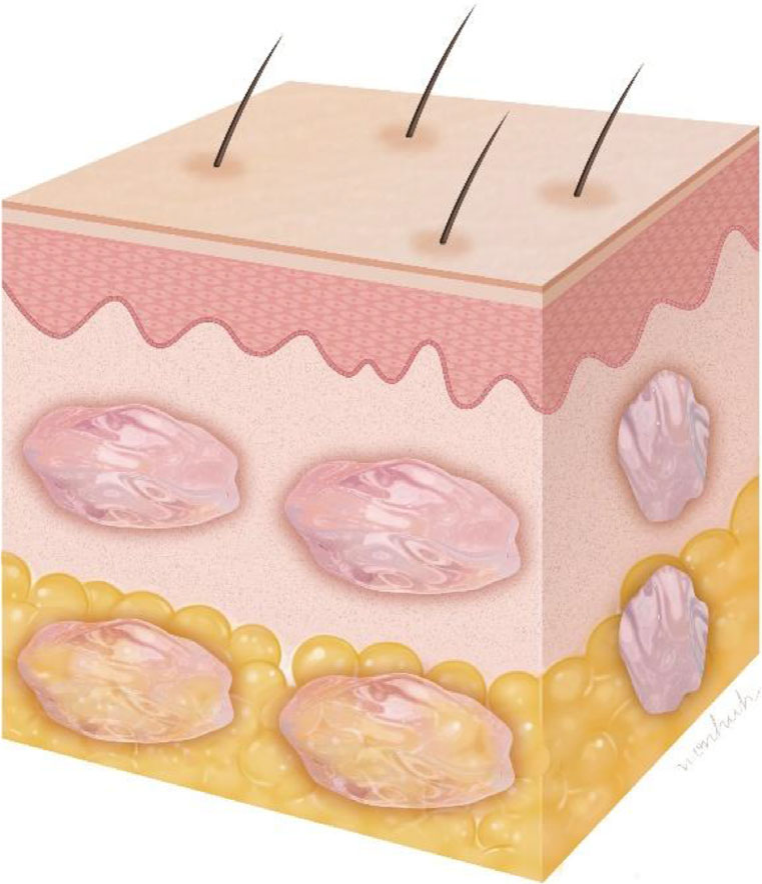
The schematic image demonstrates the injection procedure, where polynucleotide (PN) was administered to both the intradermal and subdermal layers.

The patient received a series of 1 cc PN injections (Rejuran‐s; Pharmareasearch Products, Inc., Seongnam, Republic of Korea) with a 20 mg/mL concentration. Using a 30‐gauge 8‐mm needle and intradermal puncture technique, approximately 0.02–0.03 cc of PN was injected at each point, totaling 0.3–0.4 cc near the adjacent bolus site. The remaining 0.6–0.7 cc was injected in bolus points to the superficial subcutaneous layer of the depression area (Figure 2).

**FIGURE 3:**
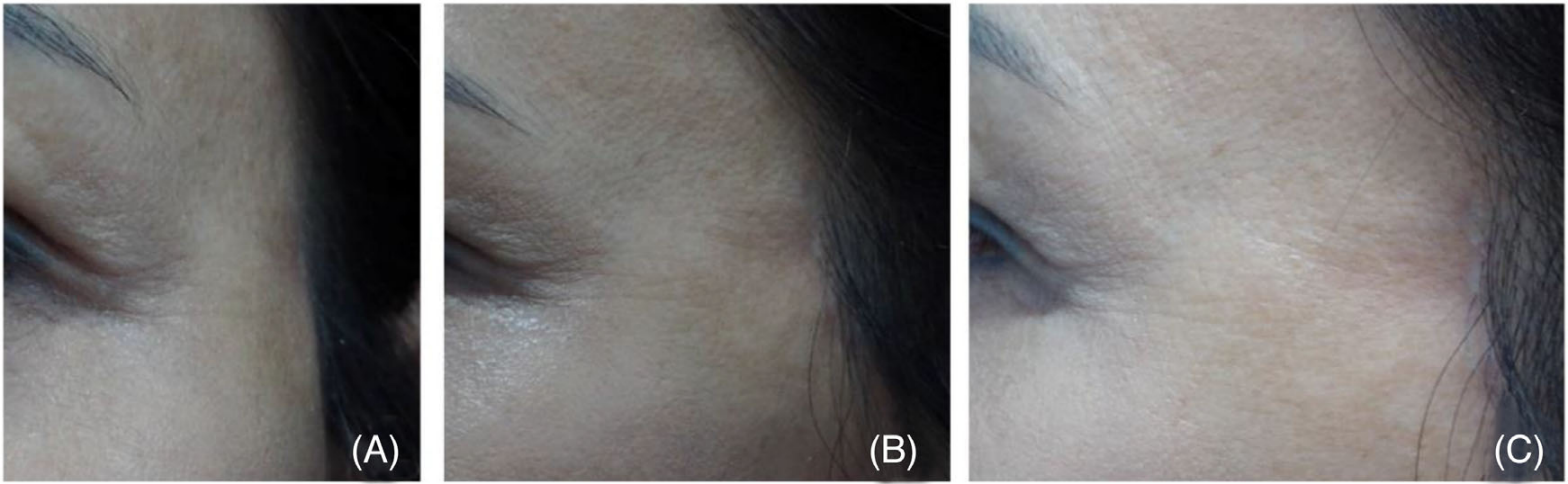
The patient received a series of six PN treatments, spaced 3 weeks apart. Notably, no significant adverse effects were observed throughout the entire treatment duration. Follow‐up conducted 2 months after the sixth treatment revealed a significant and impressive clinical improvement. Patient images are shown from frontal (A) and lateral views (B).

Four treatments were repeated at 3–4‐week intervals, without any significant adverse effects. A 1‐month follow‐up after the last treatment showed remarkable clinical improvement (Figure 3). Subsequent follow‐ups at 11 months and 21 months demonstrated maintained improvement, with the previous depression barely visible.

## 3 CASE 2

A 34‐year‐old female of South East Asian descent underwent steroid injection for two acne lesions located on her left cheek and pre‐auricular area at a different medical facility, approximately 1 week after the onset of acne. Upon presenting to our clinic 2 months after the injection, the patient exhibited two depressed areas on her left cheek.

**FIGURE 4:**
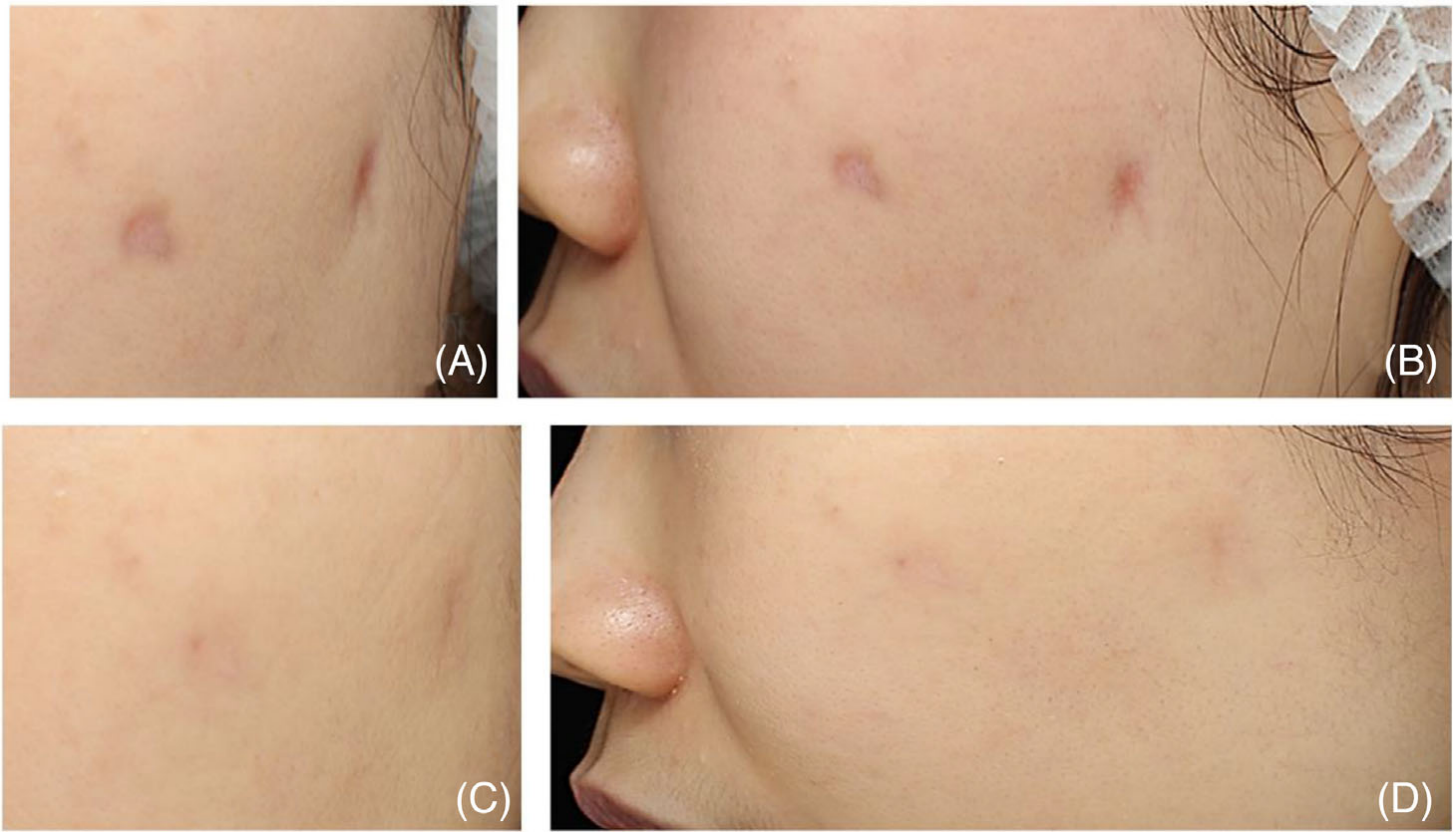
A 34‐year‐old woman received steroid injections for two acne lesions on her left cheek and left pre‐auricular area at another clinic. The depression on the left mid‐cheek was oval‐shaped and exhibited mild‐moderate erythema. The left pre‐auricular site displayed an irregular shape with two linear depressions extending distally from the center of the lesion. Patient images are shown from frontal (A) and lateral views (B). The patient underwent four treatments, with a 3−4‐week interval between each session. Importantly, no significant adverse effects were observed throughout the entire treatment period. Follow‐up conducted 2 months after treatment demonstrated a remarkable improvement in the patient's condition. Patient images are shown from frontal (C) and lateral views (D).

The left mid‐cheek depression area was oval, measuring approximately 5 × 10 mm, with mild‐moderate erythema (Figure 4). The left pre‐auricular site had an irregular shape with two linear depressions extending distally from the center, larger than the mid‐cheek lesion, and about 10 mm in diameter. The pre‐auricular depression was deeper than the mid‐cheek lesion, with more prominent erythema.

To address this concern, the patient received a series of PN filler injections (Rejuran‐s; Pharmareasearch Products, Inc., Seongnam, Republic of Korea) with a concentration of 20 mg/mL. After sterilizing the site with 0.05% chlorhexidine gluconate, the injections were administered using a 33‐gauge 4 mm needle and a serial intradermal puncture technique. Each injection point received approximately 0.01−0.02 cc of the PN product, with a total of 0.6−0.7 cc for both lesions. These injections were adjacent to each other, covering and slightly extending past the lesion margins. Additionally, approximately 0.4−0.5 cc (for both lesions) was injected in small bolus points to the superficial subcutaneous layer and slightly beyond the lesion borders, totaling 0.7 cc PN injected into both lesions per session. Four treatments were repeated at 1‐month intervals.

Throughout the entire treatment period, no notable negative effects were observed. A follow‐up conducted 2 months after the last treatment revealed a remarkable improvement in the patient's condition (Figure 4). Subsequent follow‐ups at 5 months and 12 months post‐treatment, along with gross photography images, showed almost complete healing of the previous lesions.

PN has gained considerable recognition in the realm of aesthetics, spanning from skin rejuvenation to addressing stretch marks and vulvo‐vaginal biorevitalization. Recent developments have led to the implementation of specific guidelines for its utilization.^9^, ^10^ Notably, there are no known controlled studies investigating the efficacy, safety and long‐term effectiveness of PN as a standalone treatment for soft tissue depression.

PN, a novel formulation derived from natural sources, regenerates skin with highly purified PNs at 20 mg/mL concentration, administered via dermal layer injections. High‐viscosity PN injections show potential beyond scar treatment, providing robust biomechanical responses via its 3‐D scaffold structure which can enhance clinical outcomes. The presence of a volumizing effect from injecting PN into subcutaneous layer can predict the potential efficacy including pre‐adipocyte differentiation and tightening of fibrous tissues.

In addressing iatrogenic subcutaneous fat atrophy, PN injection therapy yielded highly satisfactory outcomes with ease of use, positive patient compliance, and no significant known side effects. Patients reported high levels of satisfaction. However, larger clinical trials are needed to optimize treatment protocols and confirm efficacy. In conclusion, long‐chain PN fillers effectively fill soft tissue depressions, but more research is required to assess their safety and efficacy independently for treating volume loss.

## CONFLICT OF INTEREST STATEMENT

M.J.K., H.J.P. and S.M.O. are medical advisory board members for Pharamaresearch Co., Ltd.

## FUNDING INFORMATION

The authors received no specific funding for this work.

## ETHICS STATEMENT

This study was conducted in compliance with the principles set forth in the Declaration of Helsinki. Consent was received from the families of the deceased patients before beginning the dissections.

## Data Availability

Data are available upon request to corresponding author.
